# Diffusion and Critical Hydrogen Content of Carbon Steels with Different Strengths and Microstructures

**DOI:** 10.3390/ma19010015

**Published:** 2025-12-19

**Authors:** Dino Zwittnig, Matthias Eichinger, Martin Mülleder, Claudius Schindler, Rupert Egger, Gregor Mori

**Affiliations:** 1 Chair of General and Analytical Chemistry, Montanuniversitaet Leoben, Franz Josef-Straße 18, 8700 Leoben, Austriagregor.mori@unileoben.ac.at (G.M.); 2 Voestalpine Grobblech GmbH, Voestalpine-Strasse 3, 4020 Linz, Austria

**Keywords:** hydrogen diffusion, critical hydrogen content, carbon steel

## Abstract

Four thermomechanically rolled carbon steels with different strength levels and microstructures, namely S355M, X65M, S960M, and S1100M, were selected, and their critical hydrogen contents H_crit_ were determined. H_crit_ is the hydrogen content where brittle fracture of the steel can occur. The H_crit_ values for the four carbon steels are 2.03 (S355M), 0.91 (X65M), 0.32 (S960M), and 0.53 ppm (S1100M). Bainitic carbon steel S1100M outperforms lower-strength tempered martensitic steel S960M in terms of resistance to hydrogen embrittlement. All other steels follow the trend that a higher strength of steel results in a lower resistance to hydrogen embrittlement. The root cause for the beneficial behavior of bainitic steels is discussed.

## 1. Introduction

Hydrogen is one of the most promising energy carriers for a sustainable future. Besides the production, transport, and usage of hydrogen, hydrogen storage is one of the main challenges to overcome. At low pressures, Sieverts’ law [1,2] applies, as hydrogen can be considered as an ideal gas. At pressures above 250 bar, hydrogen can no longer be considered as an ideal gas, and in Sievert’s law, the partial pressure of hydrogen must be replaced by the fugacity f. This takes into account deviations from the ideal gas behavior resulting from the finite gas volume and interactions between the gas molecules [3]. Fugacity is dependent on the prevailing pressure and temperature, which is described by the Abel–Nobel equation. A higher temperature shifts the behavior of a gas further away from its real behavior towards its ideal behavior [4].

Besides the fugacity concept for hydrogen storage, the diffusion of hydrogen in carbon steels is important. In general, diffusion can be divided into volume diffusion (diffusion within grains), surface diffusion (diffusion at external interfaces/surfaces), and grain boundary diffusion (diffusion at internal interfaces). Volume diffusion is much slower than surface diffusion, but the amount of material transported is much larger due to the higher diffusion cross-section in grains. The significantly increased amount of material that can be transported is due to the large number of lattice planes available for volume diffusion. The diffusion cross-section for grain boundary diffusion is, like for surface diffusion, much smaller compared to volume diffusion within grains [5]. Therefore, volume diffusion is dominant over the otheitz Eltrar two.

In an ideal, completely pure iron lattice, hydrogen diffusion only occurs via interstitial lattice sites, as there are no hydrogen traps. In pure, undeformed α-iron, the diffusion coefficient for hydrogen at room temperature is in the range of 1–8 × 10^−4^ cm^2^ s^−1^ [6,7,8,9,10,11,12,13]. In the body-centered cubic lattice, hydrogen prefers tetrahedral voids as sites and only begins to occupy octahedral voids at temperatures above approximately 100 °C [7,14,15,16]. Kiuchi and McLellan described diffusion coefficients for α-iron in a temperature range from −40 to +80 °C. Their empirical description of the influence of temperature on the diffusion coefficient results in a diffusion coefficient of 7.2 × 10^−4^ cm^2^ s^−1^ at room temperature [7]. Grabke and Riecke found an expression that led to a hydrogen diffusion coefficient of 5.1 × 10^−4^ cm^2^ s^−1^ at room temperature [6]. Finally, the formulation proposed by Hagi yields a diffusion coefficient of 5.8 × 10^−4^ cm^2^ s^−1^ at room temperature [9]. Siegl carried out electrochemical hydrogen permeation measurements on single-crystal iron and ARMCO iron in various states. The diffusion coefficient for the single-crystal iron was 1.16 × 10^−4^ cm^2^ s^−1^. The ARMCO iron exhibited 1.17 × 10^−4^ cm^2^ s^−1^ in the solution-annealed state, 2.34 × 10^−5^ cm^2^ s^−1^ after 30% cold working, 6.90 × 10^−6^ cm^2^ s^−1^ after 60% cold forming, and 1.08 × 10^−7^ cm^2^ s^−1^ in the HPT-formed state (high-pressure torsion) without subsequent heat treatment [17,18]. Pure iron has a high diffusion coefficient for hydrogen and thus a high diffusion rate. The introduction of defects such as vacancies, dislocations, and grain boundaries in the metal lattice increases the trap density of the steel and lowers the effective hydrogen diffusion coefficient of the material.

Besides the above-mentioned lattice defects, foreign atoms and precipitates are responsible for trapping and for the diffusion coefficient. The higher the trap density in the alloy, especially deep traps, the lower the effective diffusion coefficient [19].

Chaudhary and Riecke investigated the influence of microstructure on hydrogen diffusion in various iron alloys. A recrystallized iron sample had the highest diffusion coefficient. For an 80% cold-formed state, the diffusion coefficient decreased by almost two orders of magnitude to 1 × 10^−6^ cm^2^ s^−1^. Martensite tempered for 2 h at 700 °C had the same diffusion coefficient for hydrogen as 80% cold-formed iron. When martensite was tempered for one hour at 500 °C, the diffusion coefficient decreased by about one quarter of an order of magnitude, and if no tempering treatment was carried out, it decreased by another quarter of an order of magnitude. A material with bainitic microstructure had a slightly lower diffusion coefficient than the non-tempered martensite of just under 1 × 10^−7^ cm^2^ s^−1^. Undeformed pearlitic material corresponded to martensite tempered at 500 °C in its diffusivity for hydrogen, and its effective diffusion coefficient gradually decreased to around 1.5 × 10^−8^ cm^2^ s^−1^ with increasing cold working degree [20,21].

According to Arrhenius’ law, the diffusion coefficient increases with temperature. Above 250 °C, most traps in steels become ineffective, and diffusion coefficients for most carbon steels are similar, independent of microstructure and trap distribution (dislocations, grain size, plastic deformation, and steel composition) [14,16,22,23,24,25].

For investigating the basic susceptibility of a material to hydrogen embrittlement, the knowledge of the critical hydrogen content is important. The critical hydrogen content H_crit_ of a steel is the hydrogen concentration in the steel above which an embrittlement due to hydrogen can occur when high plastic deformations are applied. To determine H_crit_, an in situ slow strain rate tensile test is performed, where a certain embrittlement at the surface is obtained under given charging conditions. Knowing the effective diffusion coefficient and the total hydrogen content at fracture enables the calculation of the specimen’s hydrogen diffusion profile. The critical hydrogen content can be further determined from this diffusion profile using the embrittlement depth on the fracture surface. The procedure is described in more detail in [26,27].

**Figure 1:**
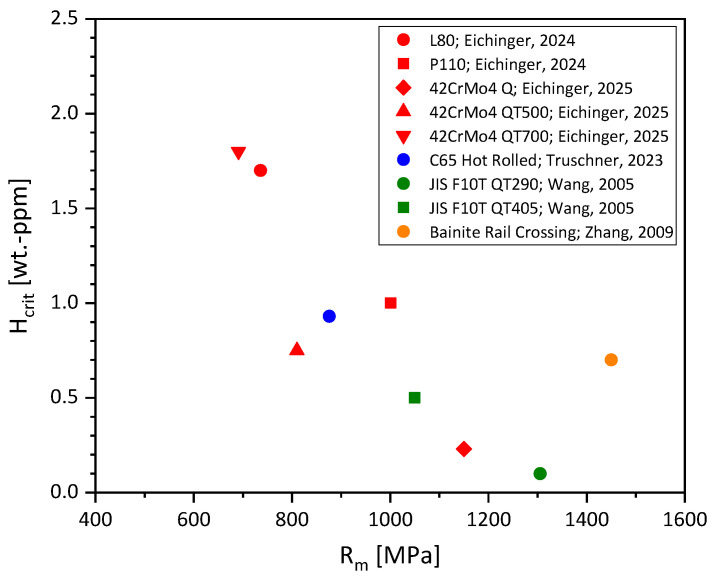
Critical hydrogen content for carbon steels from slow strain rate tensile tests as a function of ultimate tensile strength [28,29,30,31,32].

Critical hydrogen contents were collected and are compiled in Figure 1 [28,29,30,31,32]. There is a clear dependency of critical hydrogen content on ultimate tensile strength (UTS). The higher the UTS, the lower the critical hydrogen content H_crit_. Except for low-strength S355M, the strength level of the materials in Figure 1 corresponds exactly to those investigated in this work.

Pearlitic wires represent an exception to the dependency of critical hydrogen content on UTS. Truschner et al. found an increase in critical hydrogen content with a certain increasing cold deformation (equal to UTS). They explained this unusual behavior with an increasing anisotropy of the microstructure, as cold forming causes an increasing longitudinal alignment of the pearlite lamellae and a narrower lamella spacing. The fiber reinforcement effect leads to improved resistance to hydrogen embrittlement and thus to a higher critical hydrogen content for higher-strength, more cold-drawn perlites [30].

Furthermore, it is important to distinguish between the critical hydrogen content of a material (determined in this paper) and the threshold hydrogen content. While the critical hydrogen content describes the first occurrence of hydrogen embrittlement by a certain loss of ductility identifiable by SSRT testing at the highest plastic deformations, the threshold hydrogen content is the hydrogen content that, under realistic loads in practice (constant loads), results in brittle fracture. Consequently, the critical hydrogen content is the more conservative value since theoretically possible embrittlement includes severe plastic deformation of the material as well, while the threshold value gives a more practical value for smooth specimens under elastic conditions (sometimes, maybe a minor plastic deformation is present). In [33], threshold values were compiled from the literature. These hydrogen concentrations as a function of UTS are significantly higher than the H_crit_ values shown in Figure 1. Please note that in Figure 1, H_crit_ by Wang et al. [31] has been determined as the first occurrence of intergranular fracture. The authors found that for steel JIS F10T QT405 without hydrogen charging, a ductile dimple fracture occurred at 0.3 ppm, followed by a quasi-cleavage fracture and an intergranular fracture at 0.5 ppm. Therefore, a scatter bar between 0.3 and 0.5 ppm was included. For JIS F10T QT290, an immediate intergranular fracture at 0.1 ppm was observed, and thus no scatter bar was added. Zhang et al. [32] charged smooth specimens for hydrogen analysis and tensile specimens for SSRT testing in parallel. They further determined the critical hydrogen content by relating the obtained hydrogen contents to their observations during SSRT tests. Although they applied cadmium plating to prevent hydrogen losses in the tensile specimens, some hydrogen may have effused during their testing procedure. This would result in lower hydrogen contents for the tensile specimens than in the ones measured on smooth specimens and would consequently yield an overestimation of the critical hydrogen content. As the exact diffusion kinetics of the bainitic steel are unknown to the authors, no scatter bars were added.

There is still a big lack of knowledge on the effects of strength and microstructure on H_crit_. The goal of the present paper is to relate the hydrogen diffusion kinetics and the critical hydrogen content of the four investigated steel grades to their microstructural features and strength levels.

## 2. Materials and Methods

### 2.1. Materials

In this study, four different carbon steel grades were investigated. Their chemical compositions, including C, P, S, and N, were determined by optical emission spectroscopy and are shown in Table 1. All steel grades were delivered as 20 mm thick plates, being industrially manufactured based on EN 10025 [34] using a thermomechanically controlled process (TMCP).

Based on the chemical analysis given in Table 1, the carbon equivalent (*CE*) of the investigated materials can be calculated according to Equation (1):

(1) $$CE=C+\frac{Mn}{6}+\frac{Cr+Mo+V}{5}+\frac{Ni+Cu}{15}\;$$

with the elemental symbols representing the elements’ concentration (in %) in the steel [35]. Applying Equation (1) to the chemical compositions of the investigated steel grades results in carbon equivalents of 0.29 for S355M, 0.34 for X65M, 0.58 for S960M, and 0.57 for S1100M.

The mechanical properties of the investigated materials were determined via slow strain rate tensile (SSRT) tests at room temperature using a universal tensile testing machine. Non-standardized tensile specimens with a gauge length of 25 mm and a gauge diameter of 3 mm were tested using a strain rate of 1 × 10^−4^ s^−1^, controlled via the crosshead displacement. The mechanical properties yield strength (YS), ultimate tensile strength (UTS), and plastic fracture strain (A) of the four materials are displayed in Table 2.

**Figure 2:**
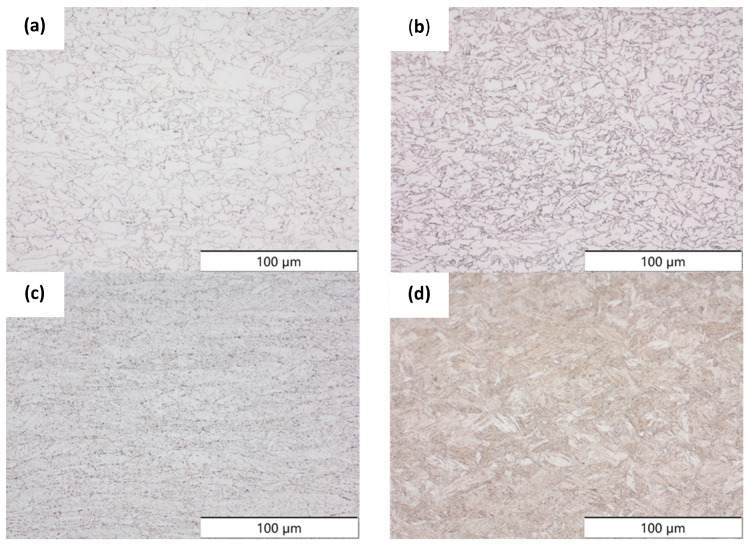
Metallographic cross-sections in the longitudinal direction of (**a**) S355M, (**b**) X65M, (**c**) S960M, and (**d**) S1100M.

To characterize the materials’ microstructures, metallographic cross-sections were prepared by grinding with SiC abrasive paper of grit sizes #120, #240, #320, #500, and #1000 and polishing with diamond paste of particle sizes 9 µm, 3 µm, and 1 µm, followed by an OPS finish. The polished cross-sections were subsequently etched using a 5% Nital solution. A light optical microscope (LOM), Olympus AX70 (Olympus K.K., Tokio, Japan), was used to record microstructural images of all investigated steel grades in the long transverse direction, which are presented in Figure 2. S355M shows a ferritic–pearlitic microstructure (Figure 2a), while X65M has a ferritic–bainitic one (Figure 2b), mainly ferrite, with a low fraction of bainite. The microstructure of S960M is tempered martensitic with little bainite, showing a discernible elongated structure in the rolling direction (Figure 2c). S1100M is a fine-grained, bainitic carbon steel (Figure 2d).

### 2.2. Hydrogen Permeation Measurement

**Figure 3:**
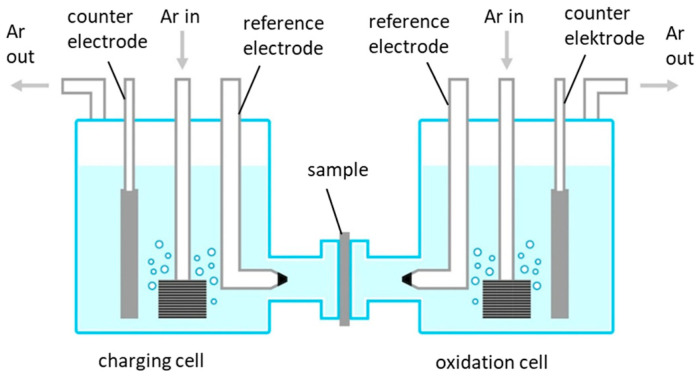
Schematic illustration of the electrochemical double cell used for electrochemical permeation measurements.

For the determination of the effective diffusion coefficient (D_eff_) for hydrogen in each material, the electrochemical permeation technique was applied. An experimental setup according to Devanathan and Stachurski [36] was used and is schematically illustrated in Figure 3. Specimens with a size of 40 × 40 mm and a thickness of 1.2 mm were manufactured from each material. Specimens were taken from a depth of 25% of the total sheet thickness to avoid testing of minor heterogeneities in the center of the sheet. The specimen’s thickness is oriented in the same metallographical direction as the thickness of the plates they were machined from, which is the short transverse direction. This corresponds to the most likely direction of hydrogen diffusion if a vessel for hydrogen storage is produced from the steel plate. The specimens were ground using SiC abrasive paper in several steps up to a grit size of #1000 on both sides and a final thickness of 1.000 ± 0.005 mm. A 100 nm thick palladium coating was applied on the oxidation side of the specimens using physical vapor deposition. In the charging compartment of the double cell, 3.5 wt.-% NaCl containing 1 gl^−1^ SC(NH_2_)_2_ was used as electrolyte. A cathodic current density of 1 mA cm^−2^ was applied for electrochemical hydrogen charging of the specimen. Regarding the oxidation compartment, a 0.1 M NaOH solution was used as electrolyte, and a constant potential of +347 mV against an Ag/AgCl electrode (+544 mV SHE) was applied. The electrolytes in both compartments were purged with Ar (99.999%) throughout the whole experiment in order to minimize the amount of dissolved oxygen as well as to ensure a constant movement of the electrolyte. The determination of the effective diffusion coefficient for hydrogen was performed using the time-lag method according to ÖNORM EN ISO 17081 [37] and Equation (2):

(2) $$D_{eff}=\frac{L^{2}}{6\;\cdot \;t_{lag}}$$

where *L* is the sample thickness, and *t_lag_* is the time that passes from the start of charging until 63% of the oxidation current’s plateau value is reached. Permeation tests were performed at room temperature and 50 °C (results for 50 °C are shown in Supplementary Materials).

### 2.3. Critical Hydrogen Content Determination

In this study, the critical hydrogen content H_crit_ is understood as the minimum hydrogen content that leads to brittle fracture in a slow strain rate tensile (SSRT) test. H_crit_ can be determined by combining the results of the SSRT test with in situ electrochemical hydrogen charging, hydrogen analysis, fractographic observations, hydrogen permeation measurements, and numerical simulation of hydrogen diffusion.

**Figure 4:**
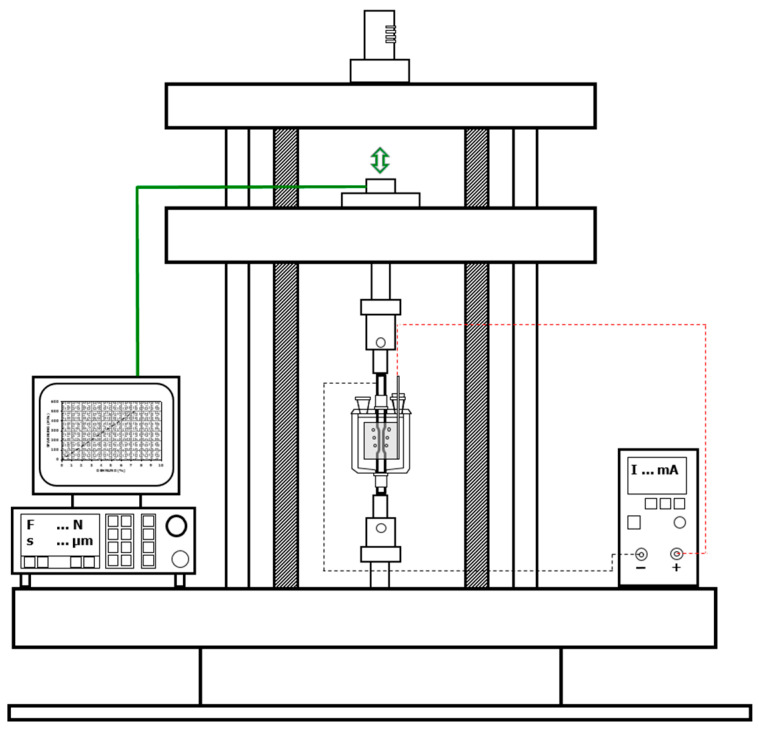
Principle of the experimental setup used for in situ charged SSRTs, the green lines represent the control of the tensile testing machine, while the green arrows depict the direction of crosshead movement. Black dotted lines show the implementation of the specimen as cathode, while red dotted lines depict the connection of a platinum mesh as anode.

SSRT tests were conducted using non-standardized tensile specimens with a gauge length diameter of 3 mm and a universal tensile testing machine whose strain rate was controlled via the crosshead displacement. The tensile specimens were taken at a similar position to the permeation specimens described in Section 2.1. In situ electrochemical hydrogen charging was performed on one specimen per material, at 25 °C, using an electrolyte of 3.5% NaCl containing 1 g L^−1^ SC(NH_2_)_2_ and charging current densities of 1 or 10 mA cm^−2^. The experimental setup is schematically depicted in Figure 4. Claeys et al. [27] showed that H_crit_ is independent of the charging duration, as long as no saturation of the specimen is reached. Therefore, H_crit_ can be interpreted as a material constant, which means that it is a value independent of the charging parameters and the used strain rate in SSRT. Furthermore, the critical hydrogen content’s character as a material constant implies its unlimited applicability in media different from the one used for the determination of H_crit_ in this study, for example, high-pressure gaseous hydrogen. The strain rate as well as the charging current density were chosen in such a way that on the fracture surface, a clearly recognizable transition from brittle to ductile fracture was achieved for each material The distance from the specimen’s surface to this transition represents the embrittlement depth (d_HE_), which was measured and averaged at three points on one fracture surface of each specimen after and in situ charged SSRT test using a Zeiss EVO MA25 (Carl Zeiss AG, Oberkochen, Germany) scanning electron microscope (SEM) with an acceleration voltage of 15 kV. Each half of the fractured specimens was removed from the experimental setup as quickly as possible after the fracture and stored in liquid nitrogen in order to maintain their total hydrogen content. Subsequently, its total hydrogen content at the time of fracture (H_fracture_) was analyzed by hot carrier gas extraction. For this purpose, a hydrogen analyzer ELTRA H500 (Eltra GmbH, Haan, Germany) equipped with a thermal conductivity cell was used. H_fracture_, the total charging time from the start of the SSRT test to the specimen’s fracture (t_fracture_), as well as *D_eff_* derived from hydrogen permeation measurements, were used to calculate a hydrogen concentration profile throughout the whole diameter of the tensile test specimen. Hydrogen concentration profiles were calculated using Fick’s second law for one-dimensional diffusion (Equation (3)) with c denoting the (hydrogen) concentration, t the time, *D_eff_* the effective diffusion coefficient, and x the distance from the surface [38].

(3) $$\frac{\partial c}{\partial t}=D_{eff}\frac{\partial^{2}c}{\partial x^{2}}$$

The solution of Equation (2) can be obtained by using the complementary error function or the application of numerical methods [27]. In the case at hand, a finite difference method with unconditional numerical stability, the Crank–Nicolson scheme, was used [38]. It was assumed that at *t* = 0, no hydrogen was present in the specimen, and in addition, Dirichlet boundary conditions were chosen because the surface hydrogen concentration is time independent. Equation (4) is the analytical solution to Fick’s second law and describes the hydrogen concentration at a certain point after a certain amount of time has passed. *C*_0_ denotes the time-independent equilibrium surface hydrogen concentration [39].

(4) $$C\left(x,t\right)=C_{0}erfc\left(\frac{x}{\bar{x}}\right)\;\mathrm{where}\;\bar{x}=2\sqrt{D_{eff}t}$$

The total hydrogen content of the specimen as a function of charging time can be described by Equation (4), where C_tot_ denotes the specimen’s total hydrogen concentration, *C_0_* the surface hydrogen concentration, *r* the radius of the charged specimen, and *x* the distance from the specimen’s surface [30]. Equation (5) is obtained by integrating the analytical solution (Equation (4)) to Fick’s second law (Equation (3)) and is used to determine the surface hydrogen concentration of the charged specimen.

(5) $$C_{tot}=\frac{C_{0}}{r}\;\left(rerfc\left(\frac{x}{\bar{x}}\right)-\frac{\bar{x}}{\sqrt{\pi }}e^{{\left(\frac{-r}{\bar{x}}\right)}^{2}}+\frac{\bar{x}}{\sqrt{\pi }}\right)\;$$

The numerical calculation procedure was performed using a code written in Python 3.9. A more detailed description of the numerical approach can be found in [30]. The obtained hydrogen concentration profile was used to determine the hydrogen content at the transition from brittle to ductile (d_HE_), which represents the critical hydrogen content H_crit_.

## 3. Results

### 3.1. Hydrogen Permeation Measurements

**Figure 5:**
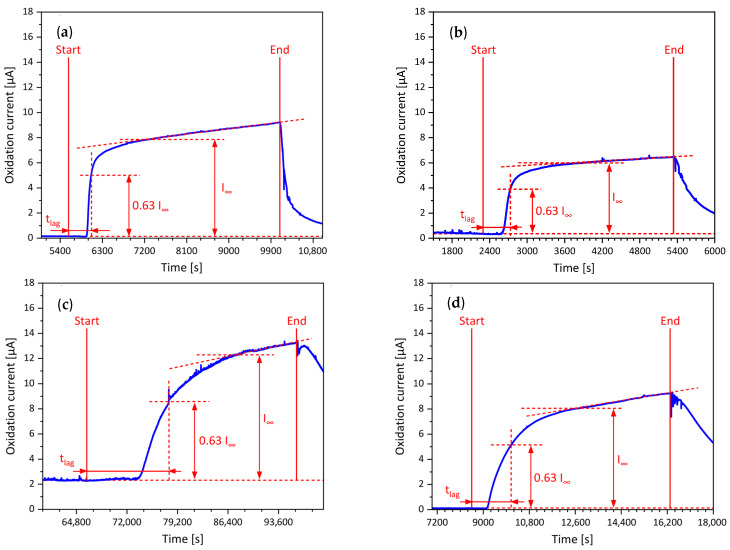
Hydrogen permeation curves at room temperature of (**a**) S355M, (**b**) X65M, (**c**) S960M, and (**d**) S1100M; the charging current density of 1 mA cm^−1^ was applied between “Start” and “End”.

To visualize the results of hydrogen permeation measurements, the oxidation currents were plotted over time, as shown in Figure 5.

The specimens were completely discharged prior to the application of the charging current. This is the reason why hydrogen charging was started at a different time for each test. None of the investigated materials showed a horizontal plateau; instead, a linear incline of the oxidation current was present. This behavior occurs due to non-constant hydrogen entry conditions at the cathodic side of the specimen, which possibly results from surface oxidation [40]. The point at which the linear incline of the measured oxidation current starts was therefore assumed to be the steady-state current I_∞_. The linear area of the curve is indicated by a dashed red line. X65M showed a smaller gradient of the linear curve area in the charged state compared to the other materials, which indicates less steady-state oxidation of the specimen’s surface.

**Figure 6:**
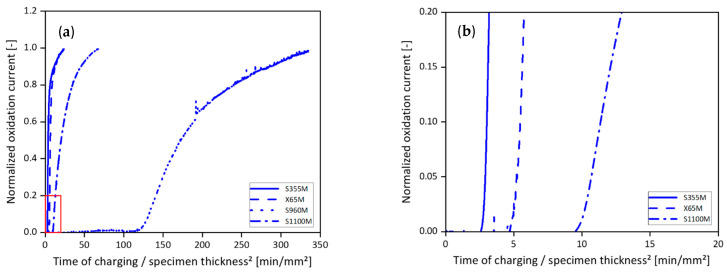
(**a**) Diffusion transients of S355M, X65M, S960M, and S1100M resulting from hydrogen permeation measurements at room temperature, and (**b**) details of the incipient increase in the permeation transients of S355M, X65M, and S1100M.

The diffusion transients shown in Figure 6 illustrate the differences in diffusion behavior between the four investigated carbon steels. S355M and X65M showed the steepest incline of the transient. S355M’s transient started to rise after approximately 2.5 min. The time passed from the start of charging until the beginning of X65M’s transient incline was just below 5 min, which is nearly double the time for S355M. According to the time-lag method described in Equation (2), the lag times of 256 s and 430 s resulted in effective diffusion coefficients of 6.46 × 10^−6^ cm^2^ s^−1^ and 3.95 × 10^−6^ cm^2^ s^−1^ for S355M and X65M, respectively. In the case of S1100M, the permeation transient had a lower slope, and the point at which it started to incline was after 10 min, double the time compared to X65M and about four times that of S355M. Hydrogen permeation measurement resulted in a lag time of 1530 s and an effective diffusion coefficient of 1.11 × 10^−6^ cm^2^ s^−1^ for S1100M. S960M showed the lowest slope. Its transient was further shifted towards long charging times and started to incline after about 120 min, roughly 12 times later than S1100M. Its measured lag time of 11,626 s corresponded to an effective diffusion coefficient of 1.45 × 10^−7^ cm^2^ s^−1^.

### 3.2. Critical Hydrogen Content Determination

**Figure 7:**
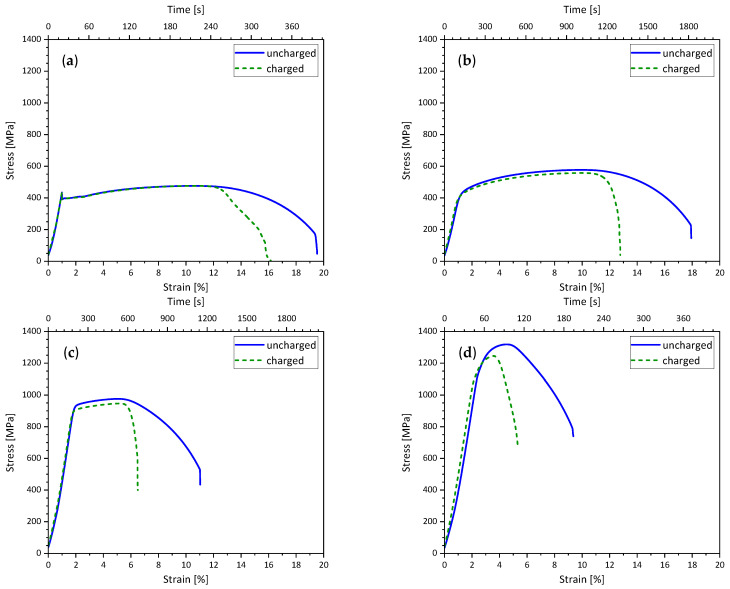
Stress–strain curves in uncharged condition (solid line) and during in situ electrochemical charging (dashed line) of (**a**) S355M, (**b**) X65M, (**c**) S960M, and (**d**) S1100M.

As SSRT tests are part of the experimental procedure to determine the critical hydrogen content, stress–strain curves of the uncharged and hydrogen-charged conditions of each material are compared in Figure 7. Due to the different strain rates used, the time from application of stress and charging current is plotted on the second *x*-axis of each diagram.

Hydrogen charging did not affect the YS and UTS of S355M. In the uncharged condition, a YS of 395 MPa and a UTS of 476 MPa were determined, while in the charged condition, a YS of 396 MPa and a UTS of 476 MPa were determined. The fracture strain A decreased from 19.4% in the uncharged condition to 16% in the charged condition. X65M showed a small decrease in YS from 439 to 423 MPa and in UTS from 577 to 557 MPa due to hydrogen charging. X65M’s fracture strain A was reduced by 28% from 17.5 to 12.6%. YS of S960M showed a decrease of 17 MPa from 918 to 901 MPa when charged with hydrogen. Its UTS of 976 MPa in the uncharged condition was reduced by 30 MPa to 946 MPa in the hydrogen-charged condition. The fracture strain A of S960M was almost halved from 10.4 to 5.6% by hydrogen charging. The effect of hydrogen charging can be noticed most clearly when looking at the highest-strength material, S1100M. YS was reduced from 1215 MPa to 1132 MPa, and UTS was reduced by 73 MPa from 1319 to 1246 MPa. Hydrogen charging caused the fracture strain to drop by 48% from 7.9 to 4.1%.

**Figure 8:**
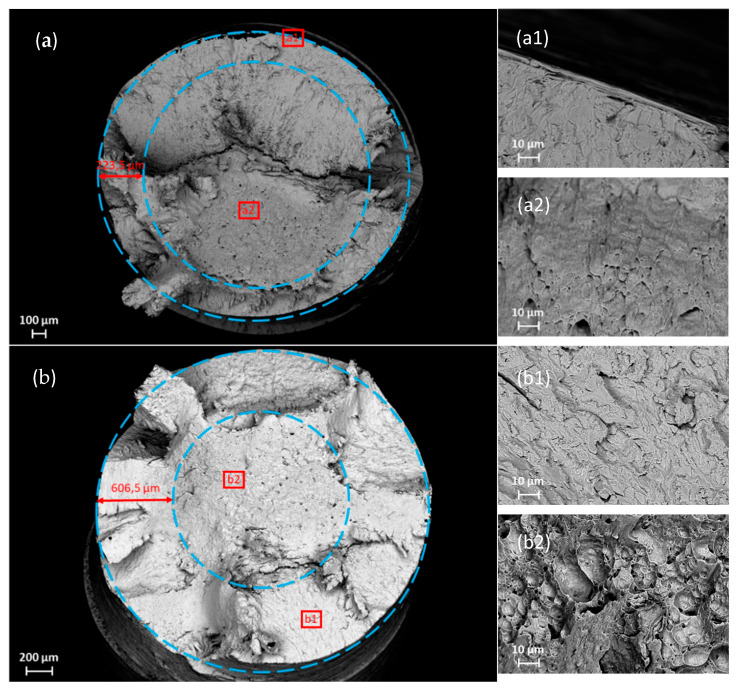

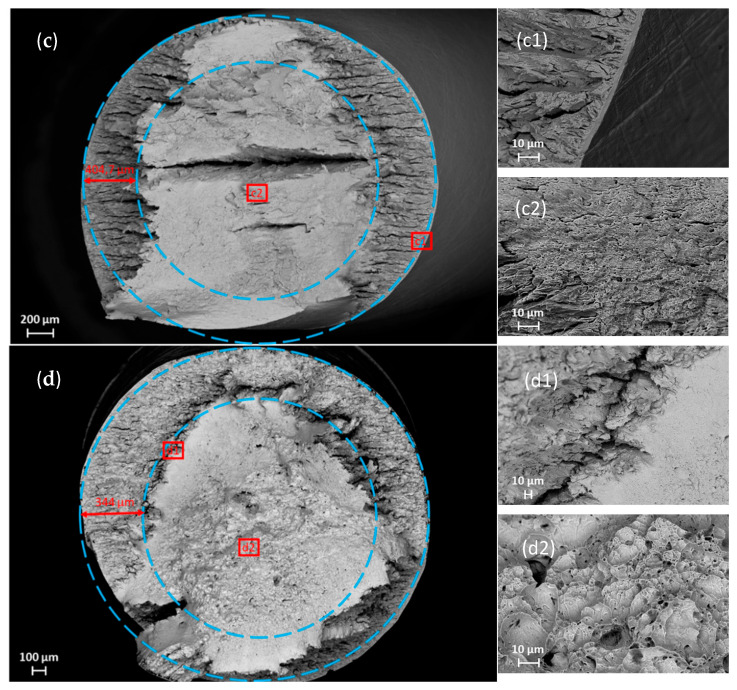
SEM pictures of the fracture surface of (**a**) S355M, (**b**) X65M, (**c**) S960M, and (**d**) S1100M; (**a1**–**d1**) and (**a2**–**d2**) represent details of the brittle and the ductile areas, respectively.

The determination of the hydrogen embrittlement depth d_HE_ was performed via SEM investigations of the fracture surface. The distance from a specimen’s surface to the transition from brittle to ductile fracture mode represents the embrittlement depth d_HE_. In Figure 8, the fracture surfaces are displayed. The two dashed blue lines represent the specimen’s surface diameter and the borderline from brittle to ductile fracture mode; the distance between them represents d_HE_. This figure also shows a detailed picture of both the brittle and ductile areas of the fracture surface of each specimen. Depending on the material’s effective diffusion coefficient, D_eff_, and charging time, d_HE_ can vary strongly. Therefore, the variation in strain rate $\dot{\varepsilon }$ and hydrogen charging current density i_charging_ were adapted in order to obtain a clearly distinguishable transition of the fracture mode.

The parameters used for the determination of H_crit_ and the results of the measurements can be found in Table 3.

**Figure 9:**
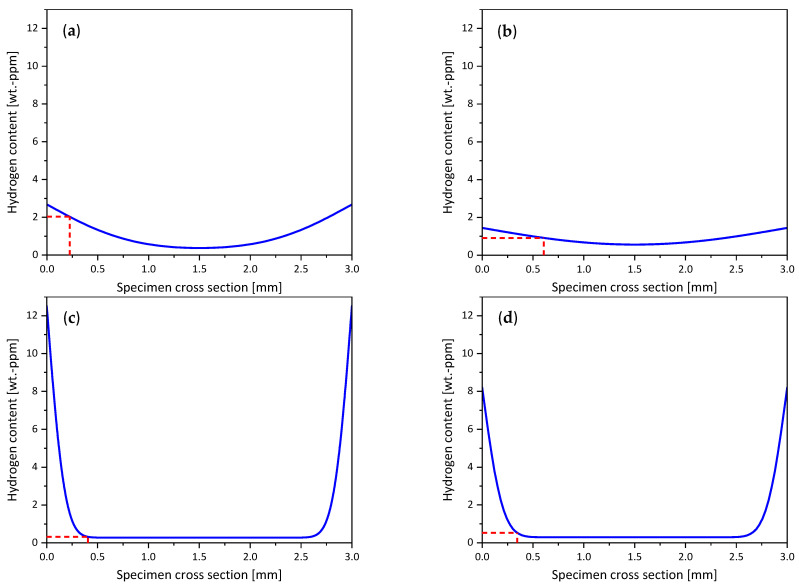
Hydrogen concentration profiles in in situ charged SSRT tests; red dashed lines represent embrittlement depth d_HE_ and critical hydrogen content H_crit_, (**a**) S355M, (**b**) X65M, (**c**) S960M, and (**d**) S1100M.

S355M was in situ charged with hydrogen during an SSRT using a current density of 10 mA cm^−2^ for a time of 329 s until the specimen fractured. The applied hydrogen charging increased the total hydrogen content of S355M from a bulk hydrogen concentration H_bulk_ of 0.27 wt.-ppm to a hydrogen concentration at fracture H_fracture_ of 0.92 wt.-ppm. The fracture surface (Figure 8a) showed a quasi-cleavage fracture mode in the outer zone (Figure 8(a1)) until a depth of 223.5 µm, where the fracture mode changed to ductile in the center of the fracture surface (Figure 8(a2)). Using the charging time, the determined effective diffusion coefficient, and the total hydrogen content of the specimen at the fracture, a hydrogen concentration profile at the time of fracture was calculated over the specimen’s cross-section (see Figure 9). One can notice that the hydrogen nearly reached the specimen’s center during the test period. Surface hydrogen concentration was calculated to be 2.7 wt.ppm. Plotting the hydrogen embrittlement depth d_HE_ (red dashed line) to the hydrogen concentration profile for S355M in Figure 9a) resulted in a critical hydrogen concentration H_crit_ of 2.03 wt.-ppm.

SSRT testing of X65M was performed using a lower strain rate of 1 × 10^−4^ s^−1^; therefore, the time until fracture t_fracture_, which is the duration of in situ hydrogen charging, was significantly longer compared to S355M, namely 1299 s. In this case, a charging current density of 1 mA cm^−2^ was chosen to achieve a proper division of brittle and ductile areas at the fracture surface. The fracture surface investigation at the SEM, illustrated in Figure 8b, showed a clearly brittle, quasi-cleavage fracture mode at the outer ring area of the fracture surface (Figure 8(b1)). At the center of the specimen’s fracture surface (Figure 8(b2)), a ductile fracture mode with large dimples was present. The line of transition from brittle to ductile fracture mode was located at a depth d_HE_ of 606.5 µm. X65M’s total hydrogen concentration was increased from H_bulk_ of 0.22 wt.-ppm to a total hydrogen concentration at fracture H_fracture_ of 0.72 wt.-ppm. The calculated hydrogen concentration profile, shown in Figure 9b, showed a flat hydrogen distribution over the cross-section of the specimen, with a surface hydrogen concentration of 1.5 wt.-ppm and hydrogen nearly reaching the center. This flat appearance of the curve is due to the relatively long hydrogen charging time. With the hydrogen embrittlement depth of 606.5 µm, a critical hydrogen content H_crit_ of 0.91 wt.-ppm was determined.

For S960M, the same parameters as for X65M in terms of strain rate and hydrogen charging current density were used for the SSRT. Fracture of the specimen occurred after a time of 678 s. The fracture surface of S960M is depicted in Figure 8c, where a linear rolling texture can be recognized. The hydrogen embrittlement depth amounted to 404.7 µm, showing a mixed brittle, quasi-cleavage fracture mode with fractions of intergranular fracture (visible in Figure 8(c1)) compared to a ductile fracture mode in the specimen’s center (Figure 8(c2)). There were pronounced secondary cracks perpendicular to the main fracture surface and parallel to the texture of the steel in the longitudinal direction. In situ electrochemical hydrogen charging augmented the total hydrogen content from 0.28 wt.-ppm bulk hydrogen to 0.93 wt.-ppm total hydrogen at fracture. Numerical calculation of the hydrogen concentration profile (Figure 9c) resulted in a steep concentration gradient and a surface hydrogen concentration of 12.5 wt.-ppm. Taking the embrittlement depth d_HE_ into account, a critical hydrogen content H_crit_ of 0.32 wt.-ppm was found.

The fractographs showed the influence of the rolling direction for S960M steel, while no significant influence was observed for the three other steel grades. Therefore, one can assume that the rolling direction is of minor importance for the results obtained for steels S355M, X65M, and S1100M. However, several secondary cracks perpendicular to the main crack plane (in the rolling direction) were observed on S960M, which indicates a certain anisotropy due to the rolling texture. For uncharged carbon steels, an expressed rolling texture results in an anisotropy of their ductility properties, which are highest in the rolling direction and lowest in the short-transversal direction. Manufacturing of tensile specimens in the short-transversal direction was not possible due to the material thickness of 20 mm; therefore, the only other possibility for specimen manufacturing other than in the rolling direction would have been in the long-transversal direction. Since the effect of the rolling texture is less pronounced in the long-transversal than in the short-transversal direction, we expect only a minor influence on the obtained critical hydrogen contents for specimens manufactured from this direction.

Strain rate $\dot{\varepsilon }$ and charging current density i_charging_ for SSRT of S1100M were increased to 5 × 10^−4^ s^−1^ and 10 mA cm^−2^, respectively. The duration of the tensile test and electrochemical charging t_fracture_ was 111 s until fracture. Like for the other three materials, a transition line between a brittle and a ductile area of the fracture surface was found. The depth to which the material was embrittled by the charged hydrogen was measured as 344.0 µm, which can be seen in Figure 8d. An embrittled, quasi-cleavage fracture mode in the outer area (Figure 8(d1)) framed the center of the specimen, where a completely ductile fracture mode was present (Figure 8(d2)). The total hydrogen content of the specimen was raised from 0.29 wt.-ppm to 0.69 wt.-ppm due to hydrogen charging. The calculated hydrogen concentration profile over the specimen’s cross-section, given in Figure 9d, showed a surface hydrogen concentration of 8 wt.-ppm, and in the edge area, a steep concentration gradient until a depth of about half a millimeter was recorded.

Figure 9 shows the calculated surface hydrogen contents from these experiments. They depend to a large extent on the hydrogen diffusion kinetics of the steels. When the hydrogen diffusion coefficient is low, but the hydrogen concentration at the time of fracture is high, as is the case for S960M steel, a high hydrogen content has to be present within a small volume fraction close to the surface, resulting in a high surface hydrogen content.

Additionally, low diffusion coefficients for hydrogen are assigned with high trapping site densities. Since the BCC lattice can only dissolve limited amounts of hydrogen, the majority of the hydrogen in carbon steels is located at trapping sites. Consequently, steels with high trap densities can take up more hydrogen compared to steels containing a low amount of hydrogen traps. This explains why the surface hydrogen contents shown in Figure 9 are low for the steels S355M and X65M (both with a low trap density), intermediate for S1100M (intermediate trap density), and high for S960M (high trap density).

The material further towards the specimen’s center is not reached by the hydrogen introduced by in situ electrochemical charging. The highlighted embrittlement depth leads to a critical hydrogen content H_crit_ of 0.53 wt.-ppm. Table 3 summarizes all the experimental parameters used for the determination of the investigated materials’ critical hydrogen content, as well as the results of each determination step.

After SEM investigations of fracture surfaces, longitudinal cross-sections through one fracture surface of each material were prepared. Table 4 shows the micrographs of all four carbon steels in different locations and magnifications. S355M steel showed several cracks starting from the outer surface, all being located in different fracture planes. The final rupture occurred as a shear fracture at an angle of 45 degrees to the axis of the tensile specimen. The detailed images at the edge and in the center of the specimen showed that no microstructural changes occurred except for some plastic deformations due to the tensile test. In the center line, some axial cracks were present due to a minor texture of the material.

Material X65M showed the same behavior as material S355M; again, there were multiple cracks at the outer surface, all in different fracture planes, and the connecting final rupture happened at 45 degrees to the specimen’s axis. Again, no microstructural changes, except plastic deformations from testing, were present.

A somewhat different appearance was found for S960M steel; there were fewer cracks at the surface. In contrast, there was a pronounced center crack due to the texture of the material. This is also described in Figure 8c. Due to the texture of the S960M steel, the surface cracks caused by early hydrogen embrittlement tended to follow the elongated microstructure in the rolling direction and were not located perpendicular to the axis of the specimen.

The highest-strength S1100M steel showed many surface cracks, which all tended to branch under an angle of 45 degrees to the original fracture plane when the embrittled zone was left.

The four carbon steels, despite some plastic deformation caused by the executed tensile tests, showed no other change in microstructure.

## 4. Discussion

### 4.1. Hydrogen Permeation Measurements

All four carbon steels show no full steady-state hydrogen flux after saturation and no true plateau current of hydrogen oxidation. The linear increase in the oxidation current in each of the four materials can be interpreted as an oxidation of the specimen’s surface during the entire experimental period [40]. Due to this, a roughening of the surface occurred, and the permeation flux increased steadily as a function of time.

**Figure 10:**
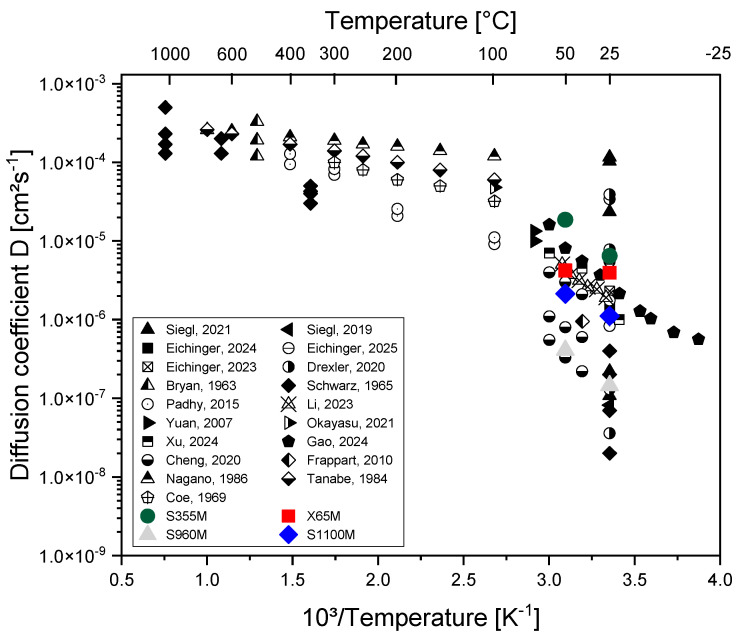
Overview of the determined effective diffusion coefficients of hydrogen in the investigated materials in comparison to other metals and alloys from the literature [17,18,19,28,29,41,42,43,44,45,46,47,48,49,50,51,52,53,54].

The determined effective diffusion coefficients of the four carbon steels at room temperature and at 50 °C are shown in Figure 10. For each material, an increase in the effective diffusion coefficient with temperature was obtained. When comparing the obtained temperature trends with the temperature-dependent data in Figure 10, there is good agreement with the expected temperature behavior. A certain scatter of permeation measurements occurred, which can easily reach a factor of 1.5 between two identical experiments. Consequently, the permeation data for steel X65M in Figure 10 show a smaller temperature dependence, while the data for S355M show a rather large temperature dependency.

Diffusion coefficients of the investigated thermomechanically treated carbon steels with different strength levels are between 1.45 × 10^−7^ and 1.1 × 10^−6^ cm^2^ s^−1^ for the two steels with high strength and tempered martensitic and bainitic microstructure (S960M and S1100M). The two steels with a high ferrite content show a higher effective diffusion coefficient between 3.95 × 10^−6^ and 6.46 × 10^−6^ cm^2^ s^−1^. This is in good agreement with data published by Riecke et al. [20,21]. The reason for the lower effective diffusion coefficient is a higher trap density for tempered martensite and bainite, which can be attributed to different amounts of precipitated carbides and the spacings of the bainite packages per se. In contrast, the highly ferritic steels S355M and X65M, due to their high ferrite content, show a higher diffusion coefficient due to a lower trap density. S355M shows the highest diffusion coefficient for hydrogen of all four carbon steels. There is a high amount of ferrite, and the perlite content in the microstructure is not sufficiently high to lower the diffusion coefficient of hydrogen substantially.

According to Chaudhary and Riecke [20], pure perlite has a lower hydrogen diffusion coefficient due to the lamella-shaped microstructure. In S355M, the perlite amount is below 5% volume fraction and cannot substantially influence the diffusion coefficient of pure ferrite.

### 4.2. Critical Hydrogen Content Determination

**Figure 11:**
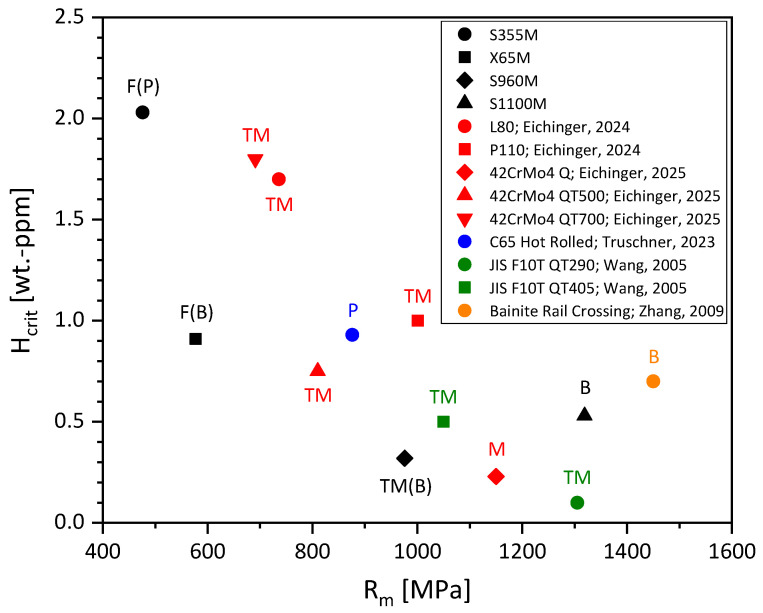
Correlation between critical hydrogen content H_crit_ and ultimate tensile strength R_m_ and microstructure of the investigated materials and data from literature [28,29,30,31,32]; F: ferrite, P: perlite, M: martensite, TM: tempered martensite, B: bainite, (…): small amount of phase.

In the second step, the critical hydrogen content H_crit_ of the four carbon steels was determined. The results are shown together with the literature data in Figure 11. At first glance, all data fit into a certain scatter band of H_crit_ from various authors. When adding the microstructure to the measurements, it is obvious that steels with mainly bainitic microstructure show a higher H_crit_ value than martensitic or tempered martensitic ones with comparable strength. Also, the pearlitic and the (mainly) ferritic steels do not change the general trend of increasing UTS on decreasing H_crit_.

The mainly ferritic steel S355M has a UTS of 476 MPa, is the lowest-strength steel among those compared based on their H_crit_ in Figure 11, and shows the highest critical hydrogen content at 2.03 wt.-ppm. In contrast, X65M, which also mainly consists of ferrite and has a comparably low UTS of 577 MPa, has a significantly lower H_crit_ of 0.91 wt.-ppm. This can be attributed to the different second phases present besides ferrite in S355M and X65M. While S355M’s microstructure shows small fractions of relatively soft pearlite at the grain boundaries, X65M exhibits small amounts of harder bainite at its grain boundaries. When strain is applied to a steel that contains hard phases within a soft matrix, the hard phases have a higher resistance to deformation than the matrix. Consequently, the matrix around those phases will deform locally much more than globally. This leads to the emission of a high amount of geometrically necessary dislocations (GNDs) around the harder phases. GNDs further represent shallow hydrogen traps, where hydrogen can accumulate, which results in a locally increased hydrogen content. This further explains, together with the general dependency of H_crit_ on UTS, why, in contrast to S355M, a substantially lower overall hydrogen content is necessary to embrittle X65M.

The material S960M, consisting of a tempered martensite, shows the lowest H_crit_ (0.32 wt.-ppm) among the investigated steel grades. Compiling this result with critical hydrogen contents of tempered martensites with similar strength levels from the literature, one can see that high-strength tempered martensites provide the lowest resistance to hydrogen embrittlement and can just tolerate minor amounts of hydrogen. Therefore, the H_crit_ value of S960M is in good agreement with literature data for steels with a comparable microstructure and strength level.

The bainitic microstructure shows higher H_crit_ values than the UTS of the steels would suggest. The higher resistance of bainite when compared to tempered martensite at or above 1000 MPa UTS is of particular interest. Tau et al. [55] also found that for AISI 4130 steel, low-temperature-tempered bainites charged with hydrogen showed a lower fatigue crack growth rate than low-temperature-tempered martensites at the same strength levels. High tempering of martensite improved the behavior of martensite significantly, while this was not the case for bainite. Therefore, after high-tempering treatment, the bainitic microstructure shows a higher fatigue crack growth rate under hydrogen when compared to tempered martensites (for equal strength levels). The authors did not give a clear reason for this temperature-dependent behavior of martensites but stated that high-temperature tempered martensite shows a spheroidized structure resulting in a mainly transgranular fracture behavior, while the higher-strength versions show a more intergranular crack propagation. For the bainitic materials, the crack propagation rate and mixed transgranular/intergranular fracture mode were independent of the strength level. In the present work, the higher-resistant bainitic S1100M showed a transgranular fracture mode, while the tempered martensitic S960M—despite lower strength—showed a mixed transgranular/intergranular fracture mode. It seems that a more transgranular fracture mode results in a higher resistance to HE.

A similar result was found by Corsinovi et al. [56]. They investigated, among other materials, a bainitic and a tempered martensitic steel grade 12.9. The bainitic failed mainly by transgranular cracking with an H_crit_ of 0.8–1.1 ppm, while the tempered martensite showed some intergranular crack paths with a significantly lower H_crit_ of 0.1–0.3 ppm. The authors explained their findings with different cracking mechanisms, with the HELP mechanism being responsible for the more transgranular bainitic material and the HEDE mechanism for the tempered martensite with the intergranular cracking. Concerning the latter, there is wide agreement that decohesion results in more grain boundary-type fractures. For the quasi-cleavage type transgranular fracture, besides HELP, the AIDE mechanism is also possible [57,58,59], which will result in exactly the same type of cracking (transgranular quasi-cleavage fracture with some very shallow dimples). A closer dislocation pile-up ahead of grain boundaries—as found by Sofronis and Robertson [60] as experimental evidence for the HELP mechanism—was neither found by Corsinovi et al. nor was it the main goal of their work. Therefore, it remains unclear whether HELP or AIDE is mainly responsible for the transgranular cracking behavior. Both mechanisms deal with dislocation movement and can explain the cracking morphology under given circumstances. A change in cracking mechanism from HEDE (intergranular crack path) to HELP/AIDE (transgranular) seems a possible explanation for these findings.

Similar results on the influence of bainite fraction in a bainite/martensite steel were published by Jo et al. [61] more recently. In their introduction, they cited older papers explaining differences between bainite and tempered martensite through differences in segregation [62], internal friction [63], and cementite morphology [64,65]. Jo et al. relate the more critical behavior of tempered martensite to a smaller portion of carbide precipitates that can act as deep hydrogen traps. Consequently, more hydrogen is present at the crack tip, the resistance to hydrogen embrittlement is reduced, and cracks propagate towards the prior austenitic grain boundaries. For a higher fraction of bainite in the lattice, there is a large number of carbide traps in the grains, and this may result in a lower hydrogen concentration ahead of the crack tip. Due to this, a less brittle behavior and a more transgranular crack path can be explained.

## 5. Conclusions

Critical hydrogen concentrations of the four investigated thermomechanically rolled carbon steels were determined as 2.03 wt.-ppm for S355M, 0.91 wt.-ppm for X65M, 0.32 wt.-ppm for S960M, and 0.53 wt.-ppm for S1100M. Further findings are as follows:

- An increasing ultimate tensile strength generally results in a decreasing critical hydrogen concentration. This is independent of microstructure as long as ferritic (S355M, X65M), pearlitic, tempered martensitic (S960M), and martensitic microstructures are considered.
- S1100M (UTS = 1319 MPa; H_crit_ = 0.53 wt.-ppm), consisting of a bainitic microstructure, shows a significantly better resistance to hydrogen embrittlement than tempered martensitic steel S960M (UTS = 976 MPa; H_crit_ = 0.32 wt.-ppm). This proves the superior resistance of high-strength bainitic materials, in contrast to tempered martensites with comparable strength levels.
- While ferritic carbon steels show a transgranular quasi-cleavage fracture, tempered martensitic steels fail intergranularly to a certain portion. In contrast, even higher-strength bainitic carbon steels show an almost pure quasi-cleavage fracture. The change in crack morphology is a sign of a change in cracking mechanism from HEDE (intergranular crack path) to HELP or AIDE (transgranular crack path).

## Figures and Tables

**Table 1:** Chemical analysis of investigated materials, in [wt.-%].

Material	C	Si	Mn	P	S	Al	Cr	Ni	Mo	Cu	V	Nb	Ti	B	N
S355M	0.03	0.35	1.39	0.01	0.001	0.04	0.13	0.01	0.00	0.00	0.00	0.03	0.01	0.00	0.005
X65M	0.03	0.34	1.59	0.01	0.001	0.05	0.18	0.01	0.01	0.01	0.01	0.04	0.01	0.00	0.004
S960M	0.08	0.33	1.61	0.01	0.001	0.03	0.61	0.03	0.48	0.01	0.07	0.01	0.01	0.001	0.006
S1100M	0.22	0.40	1.32	0.01	0.001	0.04	0.61	0.02	0.01	0.01	0.01	0.04	0.02	0.001	0.003

**Table 2:** Mechanical properties of the investigated materials.

Material	YS [MPa]	UTS [MPa]	A [%]
S355M	395	476	19.4
X65M	439	577	17.5
S960M	918	976	10.4
S1100M	1215	1319	7.9

**Table 3:** Experimental parameters and results of hydrogen permeation measurement and critical hydrogen content determination of the investigated materials.

Material	D_eff_	A	$\dot{\varepsilon }$	i_charging_	t_fracture_	d_HE_	H_bulk_	H_fracture_	H_crit_
	[cm^2^ s^−1^]	[%]	[s^−1^]	[mA cm^−2^]	[s]	[µm]	[wt.-ppm]	[wt.-ppm]	[wt.-ppm]
S355M	6.46 × 10^−6^	16.0	5 × 10^−4^	10	329	223.5	0.27	0.92	2.03
X65M	3.95 × 10^−6^	12.6	1 × 10^−4^	1	1299	606.5	0.22	0.72	0.91
S960M	1.45 × 10^−7^	5.6	1 × 10^−4^	1	678	404.7	0.28	0.93	0.32
S1100M	1.11 × 10^−6^	4.1	5 × 10^−4^	10	111	344.0	0.29	0.69	0.53

**Table 4:** Microstructure of the specimens after in situ charged SSRT tests.

Material	Overview	Detail of the Surface Near Region	Detail of the Specimen Center
S355M	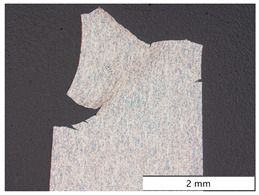	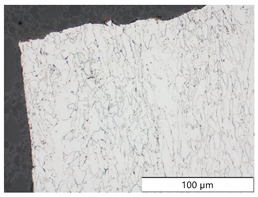	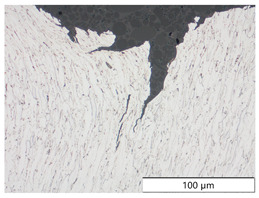
X65M	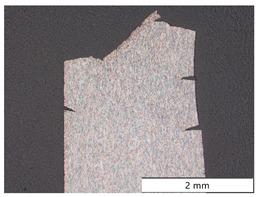	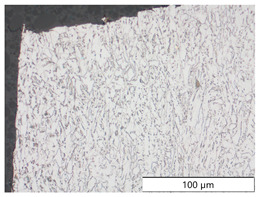	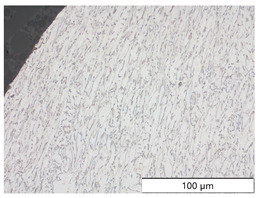
S960M	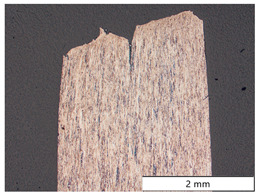	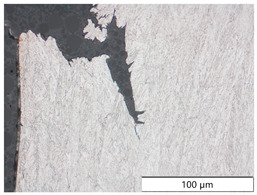	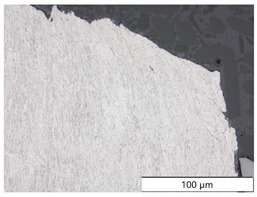
S1100M	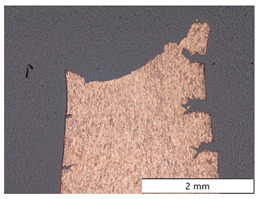	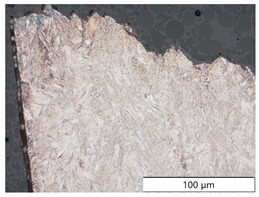	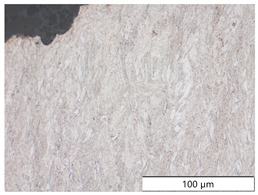

## Data Availability

The original contributions presented in this study are included in the article and Supplementary Materials. Further inquiries can be directed to the corresponding author.

## Supplementary Materials

The following supporting information can be downloaded at https://www.mdpi.com/article/10.3390/ma19010015/s1, Figure S1: Hydrogen permeation curves at 50 °C of (a) S355M, (b) X65M, (c) S960M, and (d) S1100M. The charging current density of 1 mA cm^−1^ was applied between “Start” and “End”. Figure S2: (a) Diffusion transients at 50 °C of S355M, X65M, S960M, and S1100M resulting from hydrogen permeation measurements, and (b) details of the incipient increase in the permeation transients of S355M, X65M, and S1100M.
