# Supplementary material for: Diffusion and Critical Hydrogen Content of Carbon Steels with Different Strengths and Microstructures
Source: Materials (Basel). 2025 Dec 19;19(1):15. doi: 10.3390/ma19010015 (PMC12786507; doi:10.3390/ma19010015)
Supplement: Supplementary file 1 [file materials-19-00015-s001.zip › materials-3999768-supplementary.pdf]

materials

MDPI

Supplementary Material

Diffusion and Critical Hydrogen Content of Carbon Steels with Different Strengths and Microstructures

Dino Zwittnig [1], Matthias Eichinger [1,*], Martin Mülleder [2], Claudius Schindler [2], Rupert Egger [2] and Gregor Mori [1]

[1] Montanuniversitaet Leoben, Chair of General and Analytical Chemistry, Franz Josef-Straße 18, 8700 Leoben, Austria

[2] Voestalpine Grobblech GmbH, Voestalpine-Strasse 3, 4020 Linz, Austria

* Correspondence: matthias.eichinger@unileoben.ac.at

Permeation curves of S355M, X65M, S960M, and S1100M at 50 °C

In Figure S1, hydrogen permeation curves of the four investigated materials are shown at a temperature of 50 °C.

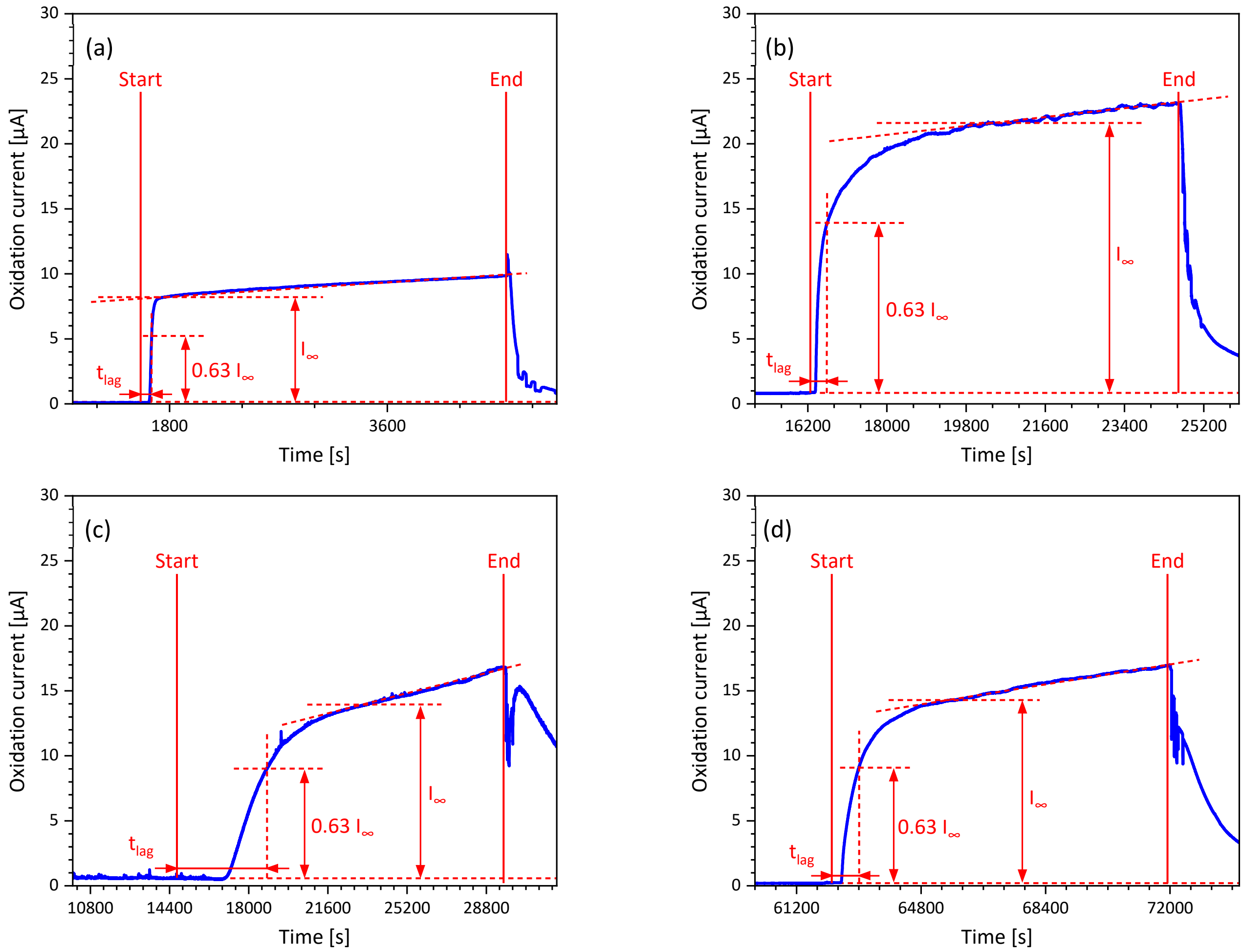

Figure S1. Hydrogen permeation curves at 50 °C of (**a**) S355M, (**b**) X65M, (**c**) S960M, and (**d**) S1100M; the charging current density of 1 mA cm^{-1} was applied between "Start" and "End".

Diffusion transients of S355M, X65M, S960M, and S1100M at 50 °C

Figure S2 shows the diffusion transients of the investigated materials at a temperature of 50 °C.

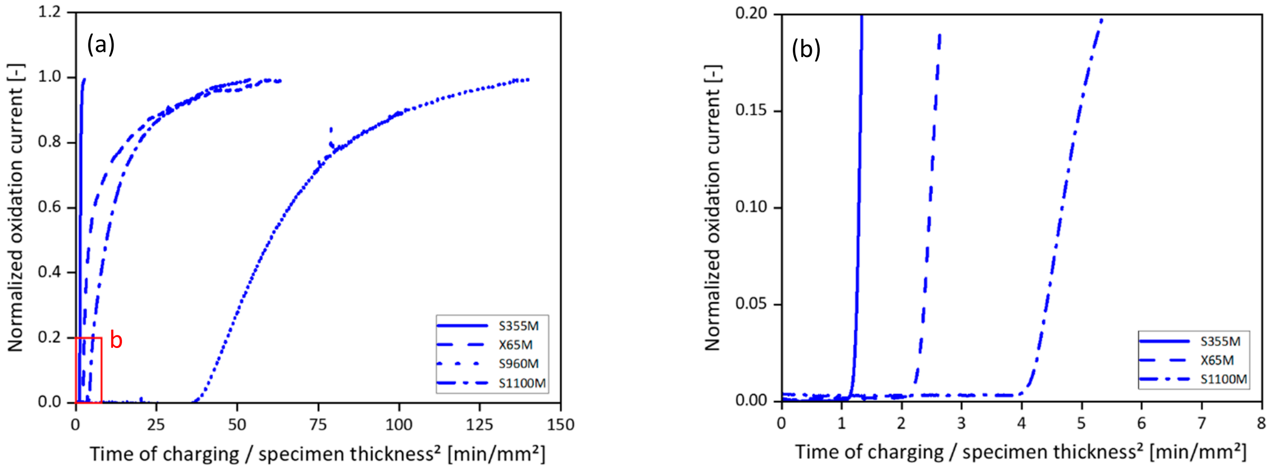

Figure S2. (**a**) Diffusion transients at 50 °C of S355M, X65M, S960M, and S1100M resulting from hydrogen permeation measurements, and (**b**) details of the incipient increase in the permeation transients of S355M, X65M, and S1100M.

https://doi.org/10.3390/ma19010015
